# Foreign Language Acquisition in Adolescent Cochlear Implant Users

**DOI:** 10.1097/AUD.0000000000001410

**Published:** 2023-07-12

**Authors:** Marita K. Everhardt, Dorit Enja Jung, Berrit Stiensma, Wander Lowie, Deniz Başkent, Anastasios Sarampalis

**Affiliations:** 1 Center for Language and Cognition Groningen, University of Groningen, Netherlands; 2 Department of Otorhinolaryngology/Head and Neck Surgery, University Medical Center Groningen, University of Groningen, Netherlands; 3 Research School of Behavioural and Cognitive Neurosciences, University of Groningen, Netherlands; 4 Department of Psychology, University of Groningen, Netherlands; 5 W.J. Kolff Institute for Biomedical Engineering and Materials Science, University Medical Center Groningen, University of Groningen, Netherlands

**Keywords:** Adolescents, Acquisition, Cochlear implants, Foreign language

## Abstract

**Objectives:**

This study explores to what degree adolescent cochlear implant (CI) users can learn a foreign language in a school setting similar to their normal-hearing (NH) peers despite the degraded auditory input.

**Design:**

A group of native Dutch adolescent CI users (age range 13 to 17 years) learning English as a foreign language at secondary school and a group of NH controls (age range 12 to 15 years) were assessed on their Dutch and English language skills using various language tasks that either relied on the processing of auditory information (i.e., listening task) or on the processing of orthographic information (i.e., reading and/or gap-fill task). The test battery also included various auditory and cognitive tasks to assess whether the auditory and cognitive functioning of the learners could explain the potential variation in language skills.

**Results:**

Results showed that adolescent CI users can learn English as a foreign language, as the English language skills of the CI users and their NH peers were comparable when assessed with reading or gap-fill tasks. However, the performance of the adolescent CI users was lower for English listening tasks. This discrepancy between task performance was not observed in their native language Dutch. The auditory tasks confirmed that the adolescent CI users had coarser temporal and spectral resolution than their NH peers, supporting the notion that the difference in foreign language listening skills may be due to a difference in auditory functioning. No differences in the cognitive functioning of the CI users and their NH peers were found that could explain the variation in the foreign language listening tasks.

**Conclusions:**

In short, acquiring a foreign language with degraded auditory input appears to affect foreign language listening skills, yet does not appear to impact foreign language skills when assessed with tasks that rely on the processing of orthographic information. CI users could take advantage of orthographic information to facilitate foreign language acquisition and potentially support the development of listening-based foreign language skills.

## INTRODUCTION

Cochlear implants (CIs) are auditory prostheses that can partially restore hearing in individuals with profound sensorineural hearing loss. An electrode array inserted in the cochlea sends electrical signals directly to the auditory nerve. This electric stimulation makes understanding spoken language possible for most device users (Blamey et al. 2013), despite the fact that the signal transmitted through the device is highly lacking in fine spectro-temporal detail (for a review, see Başkent et al. 2016b). Previous research has suggested that CI users could use cognitive mechanisms to compensate for the spectro-temporal degradations of the speech signal and, as such, enhance speech perception and language processing (Pisoni et al. 2011; Başkent et al. 2016a; O’Neill et al. 2019; Moberly et al. 2021). However, individual CI users differ in their perceptual strategies and which cognitive mechanisms they use (e.g., Benard et al. 2014; Dingemanse & Goedegebure 2019; Nagels et al. 2020). Moreover, unlike typical adult CI users, who become deaf later on in life and are implanted as adults, child CI users become deaf and are implanted early in life. As a result, child CI users need to develop speech and language skills with the spectro-temporally degraded speech signal that is inherent to electric hearing. Using this simplified binary distinction, child CI users could thus be at a disadvantage compared with adult CI users, as these children did not have the chance to develop speech and language skills with acoustic hearing. Conversely, the relatively high brain plasticity in children compared with adults could work to their advantage. In fact, some research indicates that bilaterally implanted child CI users may have higher scores in voice perception tasks and speech perception tasks with speech maskers than adult CI users, supporting such an advantage (Nagels et al. 2023). Furthermore, previous research has indicated that use of a CI facilitates native language acquisition in children, especially when implantation is done at an early age (see a recent review by Sharma et al. 2020, and for other demographic factors, see Boons et al. 2012a, 2012b). That is, the receptive and expressive native language skills in children have been shown to improve after implantation and may even reach age-appropriate levels for early implanted child CI users (Ruben 2018). To date, however, little is known about the influence of CI use on the acquisition of a non-native language during late childhood or adolescence, whether that is a non-native language that is learned as a second language (that the learner acquired and uses in daily life as they, for example, live in a country where this language is spoken) or as a foreign language (that the learner learned in an instructed setting). In young learners with normal hearing, the success of non-native language development is often ascribed to a combination of interacting factors, such as language learning aptitude, the amount and frequency of language contact, motivation, and cognitive development (for an overview, see Dörnyei & Ryan 2015). The present study explores to what degree adolescent CI users can learn a non-native language—specifically a foreign language learned in a typical school setting—similarly to their normal-hearing (NH) peers despite the degraded auditory input.

Learning a foreign or second language during late childhood or adolescence (or even adulthood) may not be the same as growing up with multiple languages from early childhood, as learning conditions and time spent on language learning tend to be crucially different (Larson-Hall 2008). The success in the language development of CI users in naturalistic bilingual settings has been assessed in a number of studies but with conflicting results. In some cases, no differences could be observed between bilingual and monolingual CI users. For example, Thomas et al. (2008) showed that early implanted age-matched child CI users from bilingual and monolingual homes in the United States performed similarly on various spoken language tasks in English. In other cases, bilingual CI users were at a disadvantage compared with their monolingual peers. For instance, Teschendorf et al. (2011) showed that the spoken German skills of early implanted school-aged CI users who grew up in bilingual homes in Germany were less advanced than those from monolingual homes. Similarly, Deriaz et al. (2014) showed that the development of spoken French skills for early implanted bilingual school-aged CI users living in the French-speaking part of Switzerland was hindered by the simultaneous acquisition of an additional language. The bilingual CI users in this study scored lower on oral language development tasks in French compared with the early implanted monolingual school-aged CI users. The results of studies comparing the language abilities of bilingual CI users with those of their bilingual NH peers also greatly varied. For example, Waltzman et al. (2003) showed that only some early implanted bilingual school-aged CI users living in the U.S. developed competency in an additional spoken language, even though the majority showed age-appropriate spoken language abilities in English. Conversely, McConkey Robbins et al. (2004) indicated that the acquisition of the additional language by early implanted bilingual child CI users mimicked that of bilingual NH children. Important to note that these studies all looked at language development in a bilingual setting, where the CI users were either exposed to or simultaneously acquired multiple languages during early childhood. Fewer studies have looked at the acquisition of an additional language learned in a foreign language classroom context during late childhood or adolescence.

To our knowledge, only a handful of studies have looked at the foreign language learning abilities of CI users during late childhood or adolescence. In a longitudinal study by Uziel et al. (2007), early implanted French 11- to 24-year-old CI users with at least 10 years of implant experience were assessed on a number of speech and language-related skills. For the assessment of foreign language skills in particular, holistic teacher ratings were included in the test battery. The CI users of this study that were still in primary or secondary school at the time of testing were rated on their English language skills by their teachers on a scale from 0 to 20, where a score of at least 10 is required of a student to advance to the next level. These holistic ratings showed that the CI users received an average score of 12.4 (±2.8), indicating that they can develop age-appropriate competency levels in a foreign language. Another study, a cohort study with Swiss German 10- to 18-year-old adolescent CI users, also explored the acquisition of a foreign language in a school setting (Beeres-Scheenstra et al. 2017). Foreign language abilities of the participants were assessed through self-assessment questionnaires and through language tasks from LinguaLevel (www.lingualevel.ch). Focusing on receptive language tasks, participants were tested on their reading and listening skills in English and French. The results showed that 64% of the tested participants performed similarly or above the Swiss school norm for English reading skills and 50% of the tested participants reached this norm for French reading skills. The participants did not perform as well in the listening tasks, with only 27% of the tested participants reaching the Swiss school norm for English listening skills and none achieving this norm for French. In a follow-up study, which included a larger cohort of 10- to 18-year-old adolescent CI users across the German-speaking part of Switzerland, a similar trend was observed (Beeres-Scheenstra et al. 2020). That is, the percentage of CI users reaching the school norms for reading skills was higher than the percentage of CI users reaching these norms for listening skills, suggesting that CI users have more difficulty performing listening tasks than reading tasks in a foreign language.

The present study assesses the foreign language skills of adolescent CI users in the Netherlands. More specifically, we investigate how well native Dutch adolescent CI users, compared with NH controls, perform in various language tasks that either rely on the processing of auditory information (i.e., listening tasks) or on the processing of orthographic information (i.e., reading and/or gap-fill task) in their native (Dutch) and their foreign (English) language. Furthermore, we investigate whether CI users’ foreign language skills may vary depending on the presentation mode of the assessment task (i.e., auditory versus orthographic). Because hearing ability is generally reduced in CI users given the spectro-temporal degradation of the signal transmitted through the device (Başkent et al. 2016b), it is likely that CI users would struggle most with assessment tasks in which audition plays a direct role. We thus expect that the adolescent CI users in this study will show lower performance in the listening tasks than in the reading or gap-fill tasks, in line with previous findings that showed that adolescent CI users can learn a foreign language (Uziel et al. 2007) but that their foreign language skills are lower in listening tasks than in reading tasks (Beeres-Scheenstra et al. 2017, 2020). To ensure that any differences observed in the foreign language skills between the adolescent CI users and their NH peers can solely be attributed to differences in hearing ability between these groups and not to cognitive differences, both groups will also be assessed on their auditory skills and cognitive skills using various tasks. On the basis of the literature, we expect that the CI users will have lower scores in the auditory tasks than their NH peers due to the degraded signal in CIs, but it is unclear how the CI users will perform on the cognitive tasks compared with their NH peers, as previous research showed mixed results (Huber & Kipman 2012; van Wieringen & Wouters 2015).

## MATERIALS AND METHODS

### Participants

Two groups took part in the study: a test group of eight early deafened and implanted 13- to 17-year-old (mean age: 15.5 ± 1.64) adolescent CI users (detailed information can be found in Table 1) and a control group of seven 12- to 15-year-old (mean age: 13.4 ± 0.94) NH adolescents. We aimed to include typically developing and well-functioning adolescent CI users who have no other health problems and who are learning English as a foreign language in a mainstream school setting, similar to their NH peers. In the Netherlands, most children start learning a foreign language in primary school, starting from approximately 8 years of age (Michel et al. 2021). All participants in this study were native Dutch learners of English in secondary school who had received at least one year of English lessons in school. The adolescent CI users had been learning English in school for 1 to 6 years (mean: 4.0 ± 1.8) and the NH adolescents for 1 to 4 years (mean: 2.6 ± 1.1). Exclusion criteria for all participants were intellectual disabilities, history of communication disorders (e.g., diagnosed with autism spectrum disorder), or neurological/psychiatric disorders other than attention deficit (hyperactivity) disorder (ADD/ADHD). Hearing ability of the NH adolescents was assessed through pure-tone audiometry at frequencies from 250 to 6000 Hz. Five NH adolescents showed audiometric thresholds ≤20 dB HL at all frequencies tested. Two NH adolescents showed audiometric thresholds ≤20 dB HL at frequencies from 1000 to 6000 Hz and thresholds between 20 and 40 dB HL at 250 and 500 Hz. These participants were still included in the study as these thresholds are very likely due to the fact that the participants were tested in their homes, instead of soundproof test rooms, and that the elevated thresholds could be reflecting the test environment rather than a hearing loss at the lower frequencies.

**TABLE 1.** Demographic characteristics of the cochlear implant (CI) users

Participant	Age (yr)	Age at Implantation (yr)	Duration of CI Use (yr)	Bilateral Implant (Yes/No)
CI1	17.0	2.5	14.5	No
CI2	16.6	1.6	15.0	No
CI3	16.8	2.4	14.4	Yes
CI4	13.8	1.8	11.9	Yes*
CI5	14.0	3.2	10.8	No
CI6	15.0	12.1	2.9	No
CI7	13.4	4.3	9.1	Yes
CI8	17.4	2.4	15.0	Yes*

published online ahead of print July 12, 2023.

* Bilateral implant, unilateral use.

CI, cochlear implant.

The study was approved by the Medical Ethical Committee of the University Medical Center Groningen (METc 2014/566). Written informed consent was obtained from all participants and their parents/guardians before participation in the study. Participants received a €20 voucher for participation.

### Language Tasks

The test battery included three easy-to-administer language tasks of varying linguistic complexity assessing language proficiency in different modalities. These tasks either relied on the processing of auditory information or on the processing of orthographic information.

#### Peabody Picture Vocabulary Task

The Peabody Picture Vocabulary Task (PPVT) is a measure of language proficiency based on receptive vocabulary skills. In this standardized task, participants were presented with a single auditory stimulus as well as four pictures and they had to select the picture that best illustrated the meaning of the word they heard. The Dutch (PPVT-NL) and English (PPVT-EN) versions of the task used in the present study were III-NL (Schlichting 2005) and IV-A (Dunn & Dunn 2007), respectively. The PPVT-NL consisted of 204 items divided over 17 sets of 12 items each and the PPVT-EN consisted of 228 items divided over 19 sets of 12 items each, where each set became progressively more difficult. Usually, the start item of the PPVT is the first item of the age-appropriate set. In the present study, to make it a bit easier for the CI users given the auditory nature of the task, the start item for PPVT-NL (for both the CI users and their NH peers) was the first item of the set that was one set lower than the age-appropriate setting. For PPVT-EN, it was not possible to determine the appropriate starting set based on the age or exposure of the native Dutch learners of English, as the PPVT-EN is designed for native speakers of English rather than learners of English as a foreign language. As such, participants all started with the first set of the PPVT-EN. Following the test protocols, participants were required to complete each set in full in sequential order. The ceiling set was the set that was completed in full where the participant made a predetermined number of mistakes. This was nine or more mistakes for PPVT-NL and eight or more mistakes for PPVT-EN. The ceiling item was the last item of the ceiling set. The remaining sets were not administered, as the PPVT was terminated after having reached the ceiling set. Unlike the test protocols, all words in this study were prerecorded. The PPVT-NL words were recorded by a female native speaker of Dutch and the PPVT-EN words were recorded by a female native speaker of (American) English.

#### C-Test

The C-test—a version of a cloze test where participants are presented with short texts in which parts of words are deleted—assesses overall language proficiency based on knowledge of grammar, vocabulary, and idioms and has been shown to correlate highly with other, more extensive language proficiency measures (e.g., Eckes & Grotjahn 2006; Harsch & Hartig 2016). The English version of the C-test used in the present study was previously used in a large cohort of adolescent native German learners of English (Nikolova 2011). This written task consisted of four English texts with a total of 111 gaps that had to be completed by the participants. Following the procedure of Keijzer (2013), participants had to complete each text within five minutes. No Dutch version of the C-test was included in the present study.

#### Sentence Verification Task

The Sentence Verification Task (SVT) tests the comprehension of both spoken language and written language as it consists of both a listening and reading condition. In this nonstandardized task designed to compare language comprehension across modalities, participants heard (i.e., in the listening condition) and read (i.e., in the reading condition) sentences that were grammatically correct, but that were either logically correct or logically incorrect. Participants had to indicate whether the sentence was logically correct or incorrect. The Dutch (SVT-NL) version of the task was adapted from Adank and Janse (2009) and the materials for the English (SVT-EN) version were adapted from sentences used in the studies by Baddeley et al. (1995) and Stringer (2014). In the present study, all 400 sentences (200 per language) were prerecorded. The 200 SVT-NL sentences were recorded by a female native speaker of Dutch and the 200 SVT-EN sentences were recorded by a female native speaker of (American) English. These were the same speakers that also recorded the PPVT words. For both the SVT-NL and SVT-EN, half of the prerecorded sentences were randomly selected for the listening condition and the remaining half for the reading condition.

### Auditory Tasks

The test battery included two auditory tasks commonly used in CI research, one assessing the temporal resolution and the other assessing the spectral resolution of the participants.

#### Gap Detection Task

The Gap Detection Task (GDT) is commonly used to measure temporal resolution in CI users (Shannon 1989; Busby & Clark 1999; van Wieringen & Wouters 1999; Cosentino et al. 2016). It is accepted to reflect the listener’s ability to perceive gaps, which is important for speech perception, as temporal gaps signal, for example, word boundaries and are useful for differentiating between phonemes. Yet, it cannot be ruled out that the change in intensity cues as a result of the introduction of the temporal gap also contributes to the gap detection thresholds, given the correlation observed between GDT and overall intensity in CI users (e.g., Shannon 1989; Garadat & Pfingst 2011), or possibly the potential changes in spectral content due to gap onset and offset. The current implementation of the GDT was adapted from Boyen et al. (2015). Participants were presented with three 500 ms filtered noise stimuli and they had to indicate which of the three stimuli differed from the other two. The noise was filtered with a bandwidth of 300 to 7000 Hz using sixth-order Butterworth filters. The stimuli were presented with 200 ms interstimulus intervals and with 50 ms onset and offset ramps. One of the stimuli was randomly selected to contain a gap with 5 ms onset and offset ramps and the gap midpoint was randomly chosen to fall between 210 and 290 ms after stimulus onset. The GDT followed a two-down/one-up adaptive procedure (Levitt 1971). The gap size was 160 ms at the start of the test and was initially adapted by a factor of 0.5. After the first reversal, the gap size was adapted by a factor of 0.707 until the end of the experiment. The GDT was terminated after eight reversals. The average of the last four reversals determined the GDT threshold. Three runs of the GDT were administered to improve the reliability of the threshold.

#### Spectral-Temporally Modulated Ripple Task

The Spectral-Temporally Modulated Ripple Task (SMRT) is a measure of spectral resolution (Henry & Turner 2003; Aronoff & Landsberger 2013; DiNino & Arenberg 2018), which is important for speech perception as it relates to the listener’s ability to perceive spectral cues in speech (e.g., vowel formants). The implementation of the SMRT was based on Aronoff and Landsberger (2013). Participants were presented with three spectrally rippled (i.e., amplitude modulated in the frequency domain) stimuli and they had to indicate which of the three stimuli differed from the other two (identical) stimuli. The target stimulus differed from the other two in the ripple density, defined by the number of ripples per octave (RPO). The SMRT followed an adaptive procedure, where the ripple density of the target stimulus was modified until participants could no longer distinguish it from the other two stimuli. These two reference stimuli had a rippled density of 20 RPO. At the start of the task, the target stimulus had a rippled density of 0.5 RPO and was adapted throughout the task with steps of 0.2 RPO using a staircase procedure. After 10 reversals, the task was terminated. The average of the last six reversals determined the SMRT threshold. Participants performed three runs of the SMRT to improve the reliability of the threshold.

### Cognitive Tasks

The test battery included three standardized and easy-to-administer cognitive tasks selected to cover a broad range of cognitive abilities, such as working memory and executive functioning.

#### Wechsler Intelligence Scale for Children III

The Wechsler Intelligence Scale for Children III (WISC-III) is a measure designed to assess the general intellectual ability of children and adolescents (Wechsler 1991). In the present study, the block design task and the picture arrangement task of the WISC-III-NL were used (Kort et al. 2005) because they do not rely on verbal information. The block design task is a timed task that measures the nonverbal problem-solving skills as well as the visuospatial and organizational processing abilities of the participants. The picture arrangement task is a timed task that provides information regarding the reasoning abilities of the participants and that relies on the ability of the participants to recognize cause-and-effect relationships.

In the block design task, participants were presented with a number of identical blocks, which had two solid white sides, two solid red sides, and two half-red and half-white sides. Using these blocks, they had to recreate a block design from an illustration. In total, there were 12 designs, which became increasingly difficult. The first design had to be recreated using two blocks, designs 2 to 9 had to be recreated using four blocks, and the last three designs had to be recreated using nine blocks. Participants had two attempts to recreate the first three designs, for the other designs they only had one attempt. The time limit for each design also increased. That is, design 1 had to be completed within 30 sec, designs 2 to 5 within 45 sec, designs 6 to 9 within 75 sec, and designs 10 to 12 within 120 sec. The age of the participants determined what design they had to start with. Following the WISC-III-NL guidelines (Kort et al. 2005), participants started with the third design.

In the picture arrangement task, participants were presented with a number of cards with illustrations. Participants had to place these cards in the correct order to construct a coherent and meaningful story. The cards were presented to the participants in a predetermined incorrect order. In total, there were 14 stories and the number of cards used to construct each story increased from three cards for the first story to six cards for the last story. Participants had two attempts to recreate the first two stories, for the other stories they only had one attempt. Like the block design task, the time limit for the picture arrangement task also increased. That is, stories 1 to 11 had to be completed within 45 sec, and stories 12 to 14 had to be completed within 60 sec. In this task, the age of the participants also determined what story they had to start with. Following the WISC-III-NL guidelines (Kort et al. 2005), participants started with the third story.

#### Corsi Block-Tapping Task

The Corsi Block-Tapping Task (CBT) is a visuospatial short-term memory task and measures working memory capacity (Corsi 1972). The task is a nonverbal alternative to the digit span task. In the present study, the digitized version of the task was used (Kessels 2012). Participants were presented with nine blocks on a screen that light up in a specific sequence and they had to indicate in what order the blocks flashed. The task started with a sequence of two blocks and the sequences gradually increased in length. Participants were presented with two sequences of the same length and they had to indicate the correct order at least once before moving on to a longer sequence, each time adding one block to the sequence length. The CBT was terminated when participants failed to indicate the correct order of a sequence of a particular length both times. The CBT block span is the length of the last sequence that was correctly completed by the participant. The highest possible block span is 9.

#### Trail Making Test

The Trail Making Test (TMT) is a visual attention task and measures mental flexibility and executive functioning (Reitan & Wolfson 1993). In this two-part task, participants had to connect a sequence of 25 dots as quickly as possible without lifting the pen from the paper. In part A, participants had to connect the numbers 1 to 25 in sequential order. In part B, participants had to alternate between numbers (1 to 13) and letters (A to L) in a sequence, connecting 1 to A, then A to 2, then 2 to B, and so on. The difference in the time taken to complete the two parts (B-A) was computed as a measure of attention and inhibition capacity.

### Procedure

Participants completed the tasks outlined above as part of a larger test battery of an exploratory study in two sessions. During the first session, participants completed the PPVT, C-test, SVT, and TMT in pseudorandomized order, counterbalancing the order of the languages of the PPVT and counterbalancing the listening and reading conditions as well as the order of the languages of the SVT between participants. During the second session, participants started with a storytelling task (not discussed here because of the focus on speech production, a concept that significantly differs from perception) and then in pseudorandomized order completed the WISC-III subtasks, CBT, GDT, a hearing screening (for NH adolescents only), the 15 words task (not discussed here as this task has both a language and a cognitive component, making it hard to place it in just one of the task categories), and ended with the SMRT. The sessions were completed either on the same day or on two different days. Each session lasted approximately two hours and participants were offered regular breaks. The sessions took place in a quiet room in the participants’ homes, using a portable test set-up of a laptop and speakers. If the sessions took place on separate days, the testing set-up between sessions was matched as closely as possible. At the start of each task, participants were provided with task-specific oral and written instructions and were given the opportunity to ask questions. Each task also started with a practice session. A pilot study before data collection with NH adolescents confirmed that participants from this age range were able to perform the tasks.

All computer-based tasks were coded and run in MATLAB (R2016a). The computer-based tasks were the PPVT, SVT, CBT, GDT, and SMRT. Auditory stimuli for these tasks were equalized in root mean square to 0.06 and calibrated to 67 dB SPL in an anechoic room. This presentation level was chosen for maximum audibility and comfort in loudness, based on preliminary informal listening evaluations by the co-authors. For the control group of NH adolescents, the presentation level was fixed throughout data collection. The test group of adolescent CI users were presented with speech and noise stimuli at the start of the first session, to confirm that auditory stimuli were audible and set at a comfortable level. If necessary, the adolescent CI users could adjust their settings (or continue using their daily settings if they wish to do so). They were instructed to keep these (adjusted) settings during the data collection and across the test sessions. The stimuli were presented in mono via a loudspeaker (the right speaker of the Creative GigaWorks T20 Series II speakers) at a distance of 70 cm.

The tasks not completed on a computer were the C-test, TMT, and WISC-III subtasks. The C-test and the TMT were completed using pen and paper and the WISC-III subtasks were completed using colored blocks and cards with illustrations. The time taken to complete a trail in the TMT and to recreate a block design and construct a story in the WISC-III subtasks was measured using a stopwatch, in line with the standardized test protocols.

## RESULTS

The data for each language task, auditory task, and cognitive task were analyzed by fitting linear models in the R environment (version 4.2.1) using the *lm* function of the *lme4* package, version 1.1-29 (Bates et al. 2015). For each task, a linear model was fitted with “group” (CI versus NH) as a predictor. Where applicable, the need for the predictor variables “language” (Dutch versus English), and “condition” (listening versus reading) was assessed through stepwise model comparison using the *anova* function, starting from the basic model with “group” as the only predictor. Post hoc analyses were performed using the *emmeans* package, version 1.7.5. (Lenth 2022). The data and code are available at https://doi.org/10.34894/LMMEVD.

### Language Tasks

#### Peabody Picture Vocabulary Task

The PPVT scoring procedure followed the guidelines of the PPVT-NL (Schlichting 2005) and PPVT-EN (Dunn & Dunn 2007). That is, the PPVT scores for each participant were calculated by taking the total number of errors made in all sets completed by the participants (starting from the starting set to the ceiling set) and subtracting that number from the number of the ceiling item. These scores were subsequently turned into the proportion of correct responses by dividing them by the total number of items for each version of the task (i.e., 204 for PPVT-NL and 228 for PPVT-EN).

**Fig. 1.**
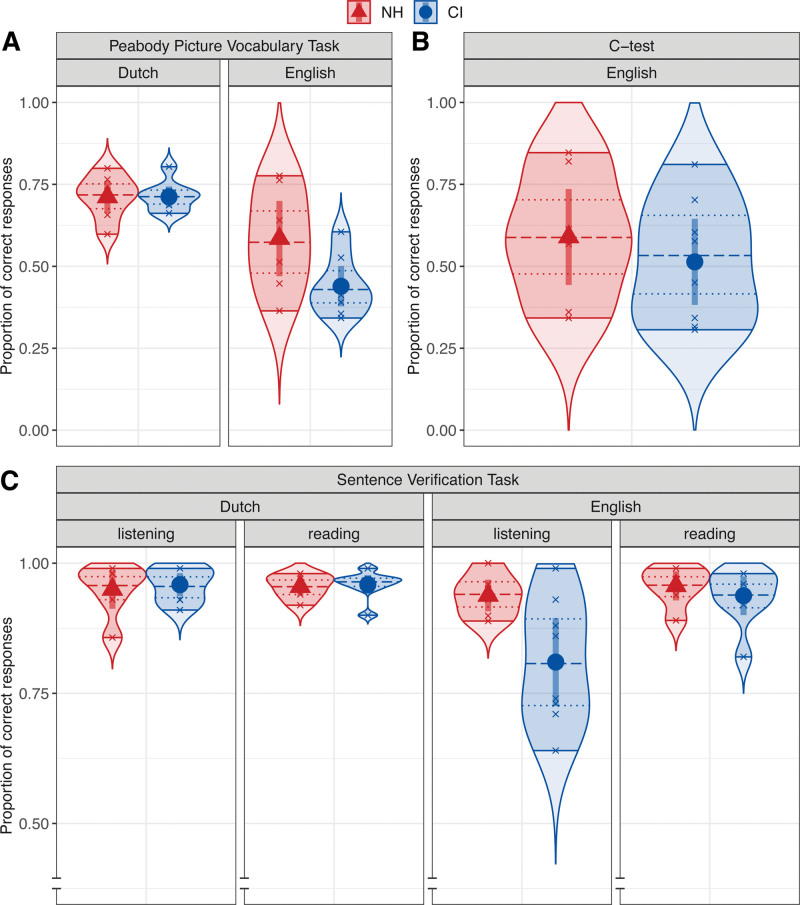
Language skills measures of the adolescent CI users (blue circles) and their NH peers (red triangles) as the mean proportion of correct responses in the Dutch and English PPVT (A), in the English C-test (B), and in the listening and reading conditions of the Dutch and English SVT (C). Error bars show the 95% confidence intervals. Smoothed density curves show the extended distribution. Shaded areas show the range. Quartile lines show the median (dashed) and the interquartile range (dotted). CI, cochlear implant; NH, normal hearing; PPVT, Peabody Picture Vocabulary Task; SVT, Sentence Verification Task.

Figure 1A shows the receptive vocabulary skills of the adolescent CI users and their NH peers in Dutch and English as the proportion of correct responses in the PPVT-NL and PPVT-EN. These results show that the proportion of correct responses was lower for English than for Dutch for both participant groups. Furthermore, the adolescent CI users and their NH peers performed similarly in Dutch, their native language, but the NH adolescents outperformed the adolescent CI users in English, their foreign language. The final linear model fitting the PPVT data as the probability of a correct response included an interaction between “group” and “language.” Post hoc pairwise comparisons confirmed that the probability of a correct response was significantly lower for English (versus Dutch) for both the adolescent CI users, *t*(26) = −5.67, *p* < 0.001, and their NH peers, *t*(26) = −2.47, *p* = 0.021. Furthermore, the response probability was significantly lower for the adolescent CI users (versus NH peers) in English, *t*(26) = −2.92, *p* = 0.007, but not in Dutch, *p* > 0.05.

#### C-Test

The C-test scoring procedure was adopted from the study by Keijzer (2013). That is, gaps left blank and gaps completed but forming an unacceptable word (e.g., grammatically or semantically incorrect) were considered incorrect and received a score of 0. Completed gaps forming the intended word or an acceptable alternative were considered correct and received a score of 1. Acceptable alternatives had to fit the context, had to be semantically and grammatically correct, and had to belong to the same word class as the intended word. Completed gaps forming the intended word containing spelling errors were also considered correct. Given the total number of gaps in this version of the C-test (Nikolova 2011), the maximum obtainable score was 111. The C-test scores for each participant were subsequently turned into the proportion of correct responses by dividing them by the maximum score.

Figure 1B shows the English C-test results for the adolescent CI users and their NH peers as the proportion of correct responses. These results show that the proportion of correct responses was slightly lower for the adolescent CI users than for their NH peers. The linear model fitting the C-test data as the probability of a correct response only included the predictor variable “group,” as this task was only performed in English. The model revealed that there was no significant difference in the probability of a correct response between the participant groups, *p* > 0.05.

#### Sentence Verification Task

Figure 1C shows the spoken language and written language comprehension skills of the adolescent CI users and their NH peers in Dutch and English as the proportion of correct responses in the listening and reading conditions of the SVT-NL and SVT-EN. These results show that the proportion of correct responses is comparable for the Dutch and English reading conditions and the Dutch listening condition, for both the adolescent CI users and their NH peers. In the English listening condition; however, a larger difference can be observed where the adolescent CI users are outperformed by their NH peers. The final linear model fitting the SVT data as the probability of a correct response included a three-way interaction between “group,” “language,” and “condition.” Post hoc pairwise comparisons confirmed that the probability of a correct response is significantly lower for the adolescent CI users (versus NH peers) in the English listening condition, *t*(48) = −4.01, *p* < 0.001, but not in the Dutch listening condition nor in the Dutch and English reading conditions, *p* > 0.05. Similarly, the response probability for the CI users was significantly lower for the English listening (versus reading) condition, *t*(48) = −4.32, *p* < 0.001, and significantly lower for the English (versus Dutch) listening condition, *t*(48) = −5.03, *p* < 0.001. None of the other pairwise comparisons reached significance, *p* > 0.05.

### Auditory Tasks

#### Gap Detection Task

**Fig. 2.**
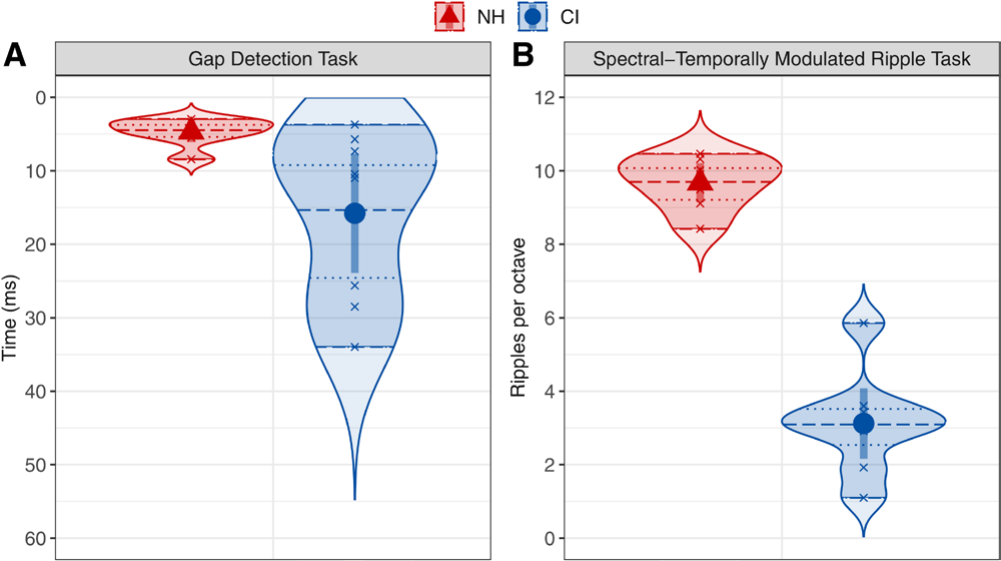
Auditory functioning measures of the adolescent CI users (blue circles) and their NH peers (red triangles) as the mean temporal resolution threshold in time (ms) for the GDT (A) and the mean spectral resolution threshold in ripples per octave for the SMRT (B). Error bars show the 95% confidence intervals. Smoothed density curves show the extended distribution. Shaded areas show the range. Quartile lines show the median (dashed) and the interquartile range (dotted). CI, cochlear implant; GDT, Gap Detection Task; NH, normal hearing; SMRT, Spectral-Temporally Modulated Ripple Task.

Figure 2A shows the temporal resolution thresholds for the adolescent CI users and their NH peers as the time (in milliseconds) in the GDT. This threshold was higher for the adolescent CI users than for their NH peers, suggesting coarser temporal resolution for the CI users. The linear model for the GDT with the predictor variable “group” confirmed that the threshold probability is significantly higher for the adolescent CI users, *t*(13) = 2.47, *p* = 0.028. The high thresholds for adolescent CI users fall within the range of thresholds for CI users found in previous research (e.g., Busby & Clark 1999).

#### Spectral-Temporally Modulated Ripple Task

Figure 2B shows the spectral resolution thresholds for the adolescent CI users and their NH peers as the number of RPO in the SMRT. This threshold was lower for the adolescent CI users than for their NH peers, suggesting a coarser spectral resolution for the CI users. The linear model for the SMRT with the predictor variable “group” confirmed that the threshold probability is significantly lower for the adolescent CI users, *t*(13) = −11.23, *p* < 0.001.

### Cognitive tasks

#### Wechsler Intelligence Scale for Children III

The WISC-III subtasks scoring procedure followed the WISC-III-NL guidelines (Kort et al. 2005). That is, scores were based on the correctness and time taken to recreate each block design or to construct each story. If a block design or picture arrangement was correctly completed within the given time limit, that design or story was given a predetermined number of points. For the block design task, this was two points for the first attempt of the first three designs and one point for the second attempt. For designs 4 to 12, this was between 4 and 7 points depending on the speed with which it was completed, with higher points given to designs completed the quickest. The maximum score for the block design task was 69. For the picture arrangement task, the number of points was two for the first attempt of the first two stories and one for the second attempt. For stories 3 to 14, the speed determined the score, with points ranging from 2 to 5. The maximum score for the picture arrangement task was 64.

**Fig. 3.**
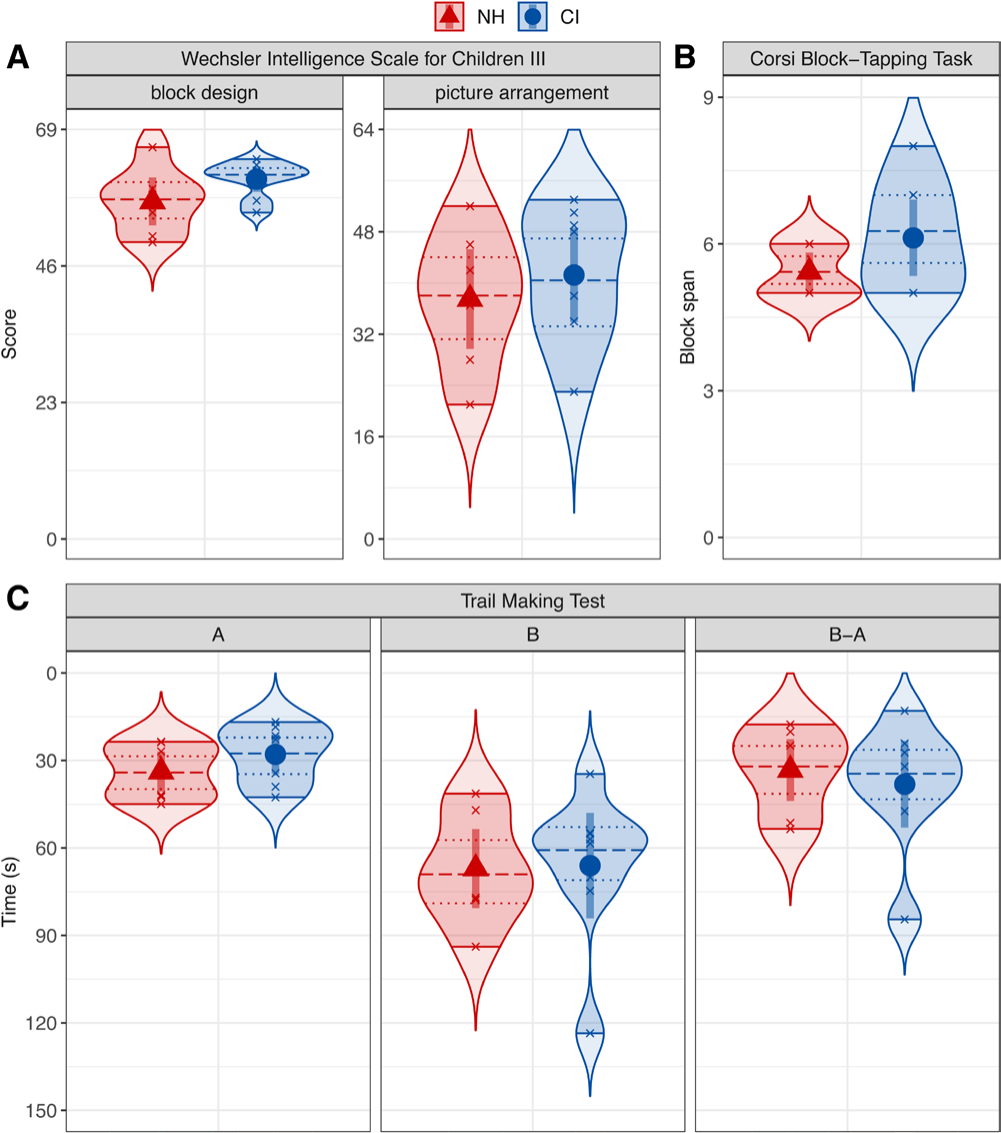
Cognitive ability measures of the adolescent CI users (blue circles) and their NH peers (red triangles) as the mean score in the block design and picture arrangement tasks of the WISC-III (A), the mean block span in the CBT (B), and the mean trail making speed in time (s) for TMT part A, TMT part B, and the difference between the two parts (B-A) of the TMT (C). Error bars show the 95% confidence intervals. Smoothed density curves show the extended distribution. Shaded areas show the range. Quartile lines show the median (dashed) and the interquartile range (dotted). CBT, Corsi Block-Tapping Task; CI, cochlear implant; NH, normal hearing; TMT, Trail Making Test; WISC-III, Wechsler Intelligence Scale for Children III.

Figure 3A shows the intellectual abilities of the adolescent CI users and their NH peers as the score in the WISC-III block design task and in the WISC-III picture arrangement task. In both the block design and the picture arrangement task, the adolescent CI users appear to outperform their NH peers. However, neither the linear model fitting the estimated WISC-III block design task score, nor the linear model fitting the estimated WISC-III picture arrangement task score, each with the predictor variable “group”, revealed a difference between the adolescent CI users and their NH peers, *p* > 0.05.

#### Corsi Block-Tapping Task

Figure 3B shows the working memory capacity of the adolescent CI users and their NH peers as the block span in the CBT. These results show that the adolescent CI users had a higher block span than their NH peers, suggesting that the working memory capacity of the CI users is greater. The linear model for the CBT with the predictor variable “group”; however, did not reveal a significant difference in the estimated block span between the participant groups, *p* > 0.05. The working memory capacity of the participants is in line with previous research that has shown that the average block span for healthy (nonbrain-damaged) participants is around six (Kessels et al. 2000).

#### Trail Making Test

Figure 3C shows the mental flexibility and executive functioning of the adolescent CI users and their NH peers as the time (in seconds) taken to complete a simple trail (A) and an alternating trail (B) in the TMT as well as the difference between these two parts (B-A). In both part A and part B of the TMT, the adolescent CI users took less time to complete a trail than their NH peers, whereas the difference between the time taken to complete the simple trails (A) and the time taken to complete the alternating trails (B) was smaller for the NH adolescents than for the adolescent CI users. Yet none of the separate linear models for these three TMT conditions, each with the predictor variable “group,” revealed a significant difference in the estimated time between the participant groups, *p* > 0.05.

## DISCUSSION

The present study used a test battery with various language, auditory, and cognitive tasks to investigate to what degree native Dutch adolescent CI users can learn English as a foreign language, whether their foreign language skills differ for listening and reading or gap-fill tasks, and whether their foreign language skills can potentially be explained by their auditory and cognitive functioning. A control group of NH native Dutch learners of English was included for comparison.

Results of the language tasks showed that native Dutch adolescent CI users can learn English as a foreign language, but the assessment of their foreign language skills provided two distinct pictures depending on the presentation mode of the task. The performance of the adolescent CI users and their NH peers was equal when assessed with reading or gap-fill tasks in the foreign language, namely the C-test (a gap-fill task) and the reading condition of the SVT-EN. However, when assessed with listening tasks, namely the PPVT-EN (a listening task) and the listening condition of the SVT-EN, the performance of the adolescent CI users was significantly lower compared with their NH peers. This is in line with previous research, which has shown that adolescent CI users are able to develop competency in a foreign language (Uziel et al. 2007), but that they generally perform lower in listening (versus reading) foreign language tasks (Beeres-Scheenstra et al. 2017, 2020). By comparison, the results of the present study for the native language tasks showed that the adolescent CI users and their NH peers performed similarly in both the reading (i.e., the reading condition of the SVT-NL) and the listening tasks (i.e., PPVT-NL and the listening condition of the SVT-NL). No matter whether they were processing auditory information or orthographic information, the native language skills of the adolescent CI users were comparable with those of their NH peers. The participant groups in this study thus appear to be quite evenly matched functionally, at least in the native language. This could be due to the fact that the CI users had on average 11.71 (±4.2) years of device experience (see Table 1), suggesting that they had sufficient auditory native language input at the time of testing, which has been shown to be important for language development (Schauwers et al. 2005). It is thus possible that with more auditory foreign language input and more time, or perhaps more tailored foreign language learning methods, the adolescent CI users will also show improved foreign language listening skills, comparable with their NH peers. Follow-up research could focus on the longitudinal effects of foreign language acquisition or on specialized foreign language training to see if the adolescent CI users can catch up with their NH peers in terms of foreign language listening skills.

The reason for including both auditory and cognitive tasks in the test battery of this study was to tease apart if any potential foreign language differences were purely due to differences in auditory functioning, as a result of the spectro-temporally degraded speech transmitted by the CI, or if there were potentially additional effects from cognitive functions. Unsurprisingly, the results of the auditory tasks showed that the auditory functioning scores were significantly lower for the adolescent CI users than for their NH peers. More specifically, the GDT thresholds showed coarser temporal resolution (acknowledging the caveats discussed in the task description above) and the SMRT thresholds showed coarser spectral resolution for the CI users when compared with their NH peers. This can be explained by the fact that the signal transmitted through a CI is highly lacking in fine spectro-temporal detail (e.g., Başkent et al. 2016b). In terms of cognitive functioning, it was unclear beforehand whether the cognitive abilities of the adolescent CI users would be lower than those of their NH peers, as previous research showed conflicting results (Huber & Kipman 2012; van Wieringen & Wouters 2015). The results of the present study revealed no significant differences between groups in any of the cognitive tasks covering a wide range of cognitive abilities, indicating that the cognitive functioning of the CI users included in this study was comparable with that of their NH peers. The comparable cognitive functioning of the adolescent CI users and their NH peers was an intended outcome of this study, as we aimed to include typically developing and well-functioning adolescent CI users who have no other health problems and who are learning English as a foreign language in a school setting, similarly to their NH peers. These adolescents were able to learn a foreign language, as evidenced by the reading and gap-fill tasks. As a result, we assume that the differences observed in the listening tasks in the foreign language can mainly be attributed to differences in auditory functioning. Future studies with larger sample sizes and less strict inclusion criteria would be helpful to investigate how generalizable the findings of the present study are to other adolescent CI users. It should be noted that given the major individual differences found in foreign language learning (see, for instance, Lowie & Verspoor 2019), this would indeed require a large sample size, which may be difficult to accomplish for this particular population.

Note that the age range of the adolescents included in this study covers the age range of secondary school students in the Netherlands, which is when they learn English as a foreign language in a school setting, up until the B2/C1 level for preuniversity students. In terms of foreign language skills, older participants with more experience are likely to outperform younger participants with less experience. A recent vocoder study covering a similar age range but where participants were divided into two distinct age groups already showed that NH adults with a mean age around 20 years outperform NH adolescents with a mean age around 13 years in a foreign language perception task with CI-simulated speech (Everhardt et al. 2022). These observations indicate that listening skills in a foreign language likely continue to develop throughout adolescence. Similarly, perceptual abilities in school-age children have been shown to continue to develop throughout childhood, but with different trajectories and with some perceptual skills not yet being adult-like by the age of 12 (e.g., Hazan & Barrett 2000; Moore et al. 2011; Koopmans et al. 2018; Nagels et al. 2020). One way to disentangle this developmental component and other individual differences among the participants would be to inspect the results across the different ages or to divide participants into groups covering smaller age ranges. The small sample size of the present study; however, prevented us from doing so. It is important to note that while the age ranges between the two groups largely overlap (i.e., both groups of participants were adolescents), there was still a small difference in these ranges. The adolescent CI users in the present study were between 13 and 17 years old and were, on average, somewhat older (mean age: 15.5 ± 1.64) than the NH adolescents (age range: 12 to 15; mean age: 13.4 ± 0.94), which could give an advantage cognitively. Similarly, the CI users had more years of experience learning English as a foreign language in school (range: 1 to 6 years; mean: 4.0 ± 1.8) than their NH peers (range: 1 to 4 years; mean: 2.6 ± 1.1), which could give an advantage in terms of foreign language skills. Hence, on a group level, despite some individual variation, the results may be indicating that the CI users may have needed additional time and experience to reach the same functional level as the NH control groups for the cognitive tasks, the native language tasks, and the foreign language tasks that relied on the processing of orthographic information. However, we acknowledge that this idea is merely speculative because the study was not designed to facilitate such a direct comparison and the sample sizes are very small, making the results sensitive to individual differences.

As a final note on the developmental aspects, the age ranges the present study has focused on is an important one. The potential developmental aspects related to this age range could have had some effects on the observed patterns. In native language development, milestones for some mechanisms, such as sensitivity to prosodic cues related to segmentation (Hirsh-Pasek et al. 1987) and sensitivity to native language phonetic sounds (Jusczyk & Thompson 1978; Kuhl et al. 2005) seem to be achieved within the very first years of childhood. Some other mechanisms seem to take a long time to develop, well into the teen years or further, especially mechanisms that require higher levels of cognitive development (Werner 2007), such as the perception of speech with speech maskers (Corbin et al. 2016; Nagels et al. 2021). Perhaps most relevant to the present study is the developmental effects related to the perception of speech degraded with vocoders (e.g., Eisenberg et al. 2000). For example, Başkent et al. (2013) showed that for the perception of pronouns and reflexives, 10- to 12-year-olds outperform younger children (5- to 8-year-olds), whereas the performance was similar to those of adults, implying this milestone is achieved by the teen years. However, when the test was repeated with vocoded materials, the adolescents were outperformed by the adults. On the basis of these findings, it could be expected that learning a foreign language, especially when combined with degradations and limitations associated with electric hearing, may take a longer time. With more experience, adolescent CI users may eventually catch up. On the other hand, children with CIs were observed to perform similarly or better than adults with CIs in some studies (e.g., Nagels et al. 2023), indicating potential advantages of CI children over adults in benefiting from their devices. These advantages have mostly been attributed to neuroplasticity (Kral & Sharma 2012), although other factors can also play a role, especially in younger implantees. For example, the use of bilateral CIs is more common in children than in adults. In our test population, we only had a few bilaterally implanted children who also use both CIs; however, they may have benefited from neuroplasticity, as most were implanted relatively young, which may help them make good use of speech sounds delivered by their implants. This is perhaps the reason for the good scores in the Dutch language tasks. Hence, from the cross-sectional data of the present study, it is not entirely possible to tease apart developmental effects and to predict how English listening skills could further improve, as developmental trajectories could take many different forms in communication disorders (Thomas et al. 2009).

In short, the cross-sectional findings of this study provide us with a general impression of foreign language learning in adolescent CI users. These findings are very positive and indicate that even with spectro-temporal degradations of the speech transmitted with a CI, adolescent CI users seem to be able to learn a foreign language in a school setting. The results also point out that it is important to choose the right modality for the assessment of foreign language skills in CI users. In terms of orthographic processing, adolescent CI users seem to perform as well as their NH peers. This is a good reflection of their robust foreign language skills. On the other hand, the same adolescent CI users show relatively lower performance in listening-based assessment, potentially implying that these adolescents may have difficulties in spoken language communication in a foreign language. If only assessed with written tasks, their potential difficulties for spoken communication in a foreign language may be overseen. Hence, an overall picture of the abilities and difficulties adolescent CI users may have with foreign language learning is perhaps only possible with a comprehensive assessment that relies on both reading and writing tasks, as well as listening and conversation-based tasks.

## CONCLUSIONS

This study explored to what degree adolescent CI users can learn a foreign language despite the degraded auditory input. The results confirmed that CI users can develop competency in a foreign language, yet the assessment of their foreign language skills led to distinct results for, on the one hand, the listening tasks and, on the other hand, the reading and gap-fill tasks. The group of native Dutch CI users learning English as a foreign language in this study showed foreign language abilities comparable with their NH peers for the English reading and gap-fill tasks, but showed significantly lower performance in the English listening tasks. This discrepancy in performance between auditory and orthographic language tasks was only observed for the foreign language. In the native language, the adolescent CI users and their NH peers performed similarly no matter the presentation mode of the assessment task. Furthermore, no differences in the cognitive functioning between the CI users and their NH peers were found that could explain their foreign language abilities. Taken together, the results of this study show that CI users that acquire a foreign language with degraded auditory input are at a disadvantage compared with their NH peers that acquire a foreign language with normal (nondegraded) auditory input, but only when looking at listening skills in the foreign language. The CI population could thus benefit from additional support for the development of listening-based foreign language skills.

## ACKNOWLEDGMENTS

This research was supported by the Center for Language and Cognition Groningen, the Netherlands Organization for Scientific Research and the Netherlands Organization for Health Research and Development [VIDI 016.096.397; VICI 918-17-603], and the Heinsius Houbolt Foundation.
